# Management of brain metastases with stereotactic radiosurgery alone versus whole brain irradiation alone versus both

**DOI:** 10.1186/1748-717X-9-116

**Published:** 2014-05-20

**Authors:** Mahmoud M El Gantery, Hoda M Abd El Baky, Hesham A El Hossieny, Mohamed Mahmoud, Osama Youssef

**Affiliations:** 1 The Department of Radiation Oncology, National Cancer Institute, Cairo University, Cairo, Egypt

**Keywords:** Whole brain irradiation, Stereotaxy, Radiotherapy, Brain metastases

## Abstract

**Introduction:**

This prospective randomized study aimed to evaluate the role of WBRT + SRS compared to SRS alone and to WBRT alone in improvement of overall survival, brain local control and neurologic manifestations.

**Patients and methods:**

The trial included 60 patients with 1 to 3 brain metastases treated at the Radiotherapy Department, National Cancer Institute. 21 patients received WBRT + SRS, 18 patients received SRS alone and 21 patients received WBRT alone.

**Results:**

Median local control was significantly better for WBRT + SRS compared to SRS alone & WBRT alone (10 vs 6 vs 5 months, respectively, P = 0.04). There was non significant survival benefit for WBRT + SRS compared to SRS alone & WBRT alone. Survival was significantly better for patients with controlled primary tumor who received WBRT + SRS compared to SRS alone & WBRT alone (median survival was 12 vs 5.5 vs 8 months, respectively. P = 0.027). Regardless of the treatment group, median survival and median local control were highly significantly better for single brain site involvement compared to multiple brain sites involvement (P = 0.003 & P = 0.001, respectively), and median brain local control was significantly better for single lesion compared to multiple lesions (P = 0.05).

**Conclusions:**

WBRT + SRS is an effective, safe tool in treatment of patients with 1 to 3 brain metastses improving the brain local control, but further studies with larger number of patients is recommended.

## Introduction

Brain metastases are the most common intracranial tumors in adults, accounting for more than one-half of brain tumors. In patients with systemic malignancies, brain metastases occur in 10%-30% of adults and 6%-10% of children [1-3]. The incidence of brain metastases appears to be rising as a result of superior imaging modalities and earlier detection [4].

The most common route of metastatic dissemination resulting in brain metastases is hematogenous, and it is therefore presumed that the entire brain is “seeded” with micrometastatic disease, even when only a single intracranial lesion is detected [5]. Recently, this assumption has been questioned, prompting a contrarian philosophy that in some patients, the intracranial disease is truly limited—the so-called oligometastases situation [5].

The management of brain metastases can be divided into symptomatic and therapeutic strategies. Symptomatic therapy often includes corticosteroids to reduce peritumoral edema and anticonvulsants to prevent recurrent seizures. Therapeutic approaches to brain metastases include surgery, whole brain radiotherapy (WBRT), stereotactic radiosurgery (SRS), and chemotherapy. Many patients are treated with a combination of these, and treatment decisions must take into account factors such as patient age, functional status, primary tumor type, extent of extracranial disease, prior therapies, and number of intracranial lesions [4].

WBRT has been used for decades as the mainstay of the management of patients with brain metastases being widely applicable and technically easy to deliver. It also improves symptoms in 75% of symptomatic patients with brain metastases and improves median survival to 3–6 months compared with a median survival of only 1–2 months in untreated patients [6,7].

However, for patients who truly have limited intracranial disease, the potential exists that WBRT could be replaced by focal therapeutic options such as resection or SRS [5].

Also, the recognition of potential late neurotoxicity and the inadequate local control with WBRT and the development of better imaging and focal therapies, such as microsurgical resection and SRS, have shifted the research focus to study which patients are best treated with WBRT and which patients are best treated with other therapies. Numerous randomized trials have investigated strategies for patients who would most likely benefit from these aggressive therapies, namely those of good performance status with single or few brain metastases. The median survival times reported for these relatively favourable prognosis patients enrolled in these trials were typically 6–11 months [8].

SRS employs multiple convergent beams to deliver a single, large dose of radiation to a discrete target volume. The three most common delivery systems are the linear accelerator, gamma knife, and cyclotron, which make use of high-energy photons, gamma rays, and protons, respectively. Most brain metastases have distinct radiographic and pathologic margins, making them attractive targets for SRS [4]. SRS has emerged as a common treatment modality for newly diagnosed patients, alone or in combination with WBRT, and as salvage therapy for progressive intracranial disease after WBRT.

### Aim of the study

The current study is a prospective randomized study to evaluate role of WBRT combined with SRS compared to SRS alone and to WBRT alone in improvement of the overall survival, brain local control, performance status and its effect on treatment-related morbidity.

### Patients and methods

The present work involved 60 patients with 1 to 3 brain metastases, each with a maximum diameter of no more than 4 cm on contrast-enhanced MRI scans, derived from a histologically confirmed systemic cancer. An ethical approval and an informed written consent was obtained from the patients before entering the study. This study was approved by the ethical committee of National Cancer Institute Cairo University Egypt.

These patients are randomized into 3 arms, 21 patients received whole brain radiotherapy WBRT, 18 patients received SRS and 21 patients received WBRT + SRS, the study took place in the radiotherapy department, National Cancer Institute, Cairo University, Egypt from January 2008 until March 2011 with the following inclusion criteria: Age ≤ 70 years, KPS ≥ 70%, Ensured adequate organ function (Haemogram, Kidney and Liver function), no previous treatment for brain metastases.

### Regarding technique of treatment

1) **Patient immobilization**

Head and neck immobilization for patients of WBRT group was performed using thermoplastic mask and head support in supine position.

**Figure 1:**
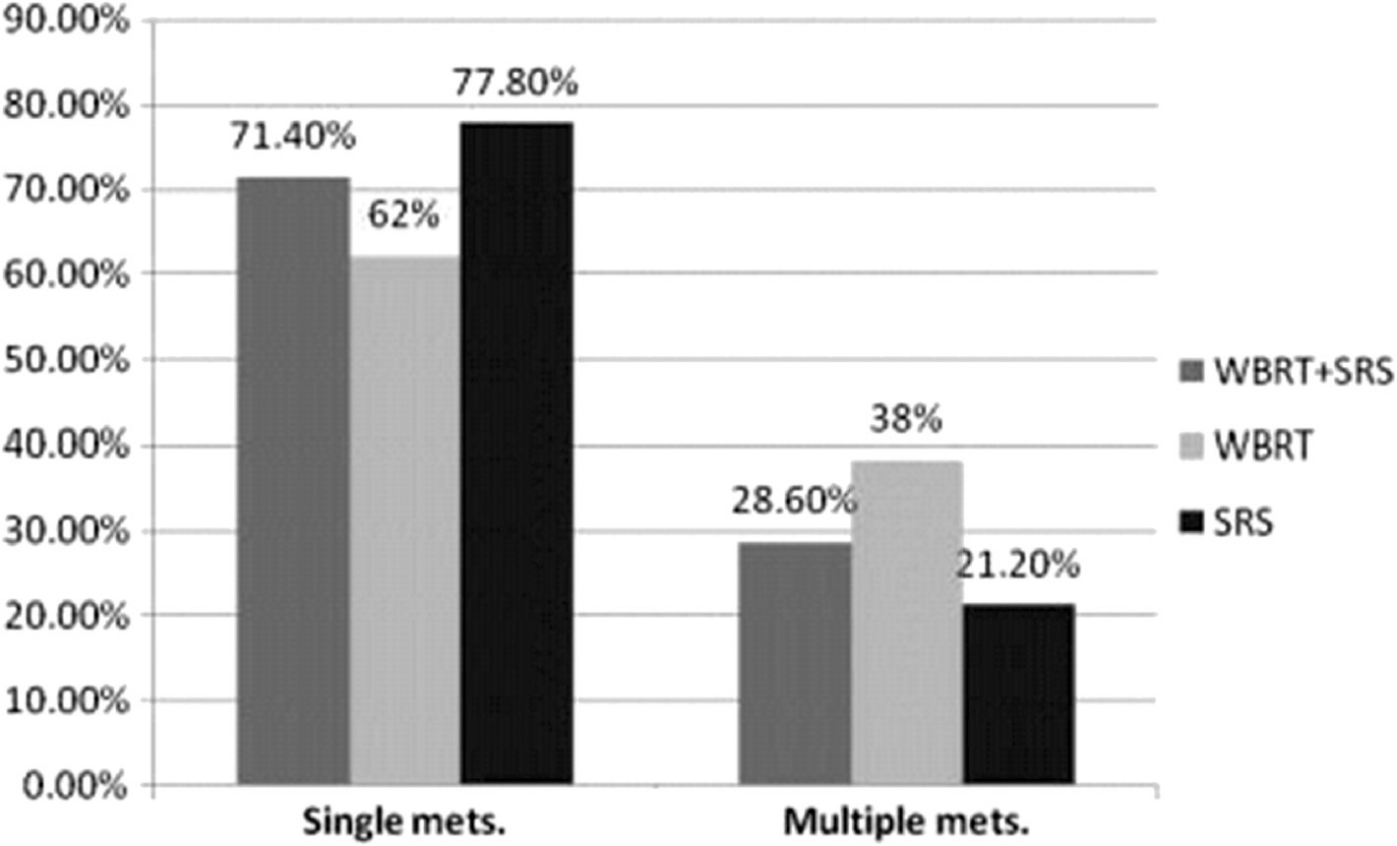
Distribution of patients in studied arms according to no. of brain lesions.

Precise immobilization of patients of the SRS group was done by the Brown-Roberts-Wells CT Stereotactic (BRW) localization system frame. Local anaesthesia (1 cc of 0.5% xylocaine) was injected subcutaneously at the site of insertion of each headpins; the pins are artifact free carbon fiber with ceramic tips as shown in Figure 1.

2) **Image acquisition** (**CT- MRI- Fusion)**

The MRI study consists of post-contrast T1-weighted images, followed by the acquisition T2-weighted and FLAIR images. Slice thickness was 2.5 mm with no gaps.

For patients receiving SRS; a Brown-Roberts-Wells CT stereotactic (BRW) frame and localizer system were used and attached to the CT couch, using an adjustable support bracket. CT scan was taken throughout the full cranial volume from the superior edge of the frame to the cranial vertex after injection of intra venous contrast. The image slice interval was 2.5 mm. The patient’s images were transferred to the treatment planning computer using the transfer protocol and optical disc.

Axial MRI images were fused to the axial CT images using chamfer match technique where sets of points belonging to the same anatomical structures on each study were used.

3) **Target and critical organs deleneation**

The stereotactic team began by delineating on each image slice the external cranial surface, dose limiting structures such as optic nerves and chiasma, eyes, functional brain areas such as the brainstem and the target volume which included the gross target volume +1 mm as planning target volume. In addition, all nine rod intersections on each slice are digitized to define the transformation for each slice from the CT reference system to the frame system.

4) ***Selection of method of treatment***

The most important criteria for selection of SRS method were tumor volume and shape. Patients with radiographically well defined lesion with small volume were candidate for SRS using non coplanar arcs by circular collimator (cones). In general, arc therapy was preferred in tumors with rounded regular surface, while lesions with large volume and irregular outlines were treated using mMLC.

5) **Evaluation of the treatment plans**

Multiple parameters were evaluated including surface dose, isodose distribution, dose volume histogram, conformity index and tissue volume ratio.

6) **Quality assurance**

To confirm the precision of isocenter, as SRS treatment uses the rotation of gantry and couch. Mechanical Isocenter Standard (MIS; Radionics Inc.), Rectilinear Phantom Pointer (RLPP; Radionics Inc.) were used to achieve this precision.

7) **Dose specification and fractionation**

The WBRT dosage schedule is 30 Gy in 10 fractions over 2 weeks delivered using megavoltage machines with photon beams of energy 6 MV. Treatments were delivered through parallel opposed fields that cover the entire cranial contents. Doses were specified at the central axis at midplane on the brain.

The WBRT treatment preceded SRS when patients were assigned to the WBRT + SRS group and the whole treatment duration was within 1 month.

In the current study, the prescribed dose of SRS in the WBRT + SRS arm ranged from 14 to 20 Gy (mean = 14.6 Gy, median = 14 Gy), while the prescribed dose in the SRS alone arm ranged from 18 to 20 Gy (mean = 19.5 Gy, median dose = 20 Gy). The dose choice was dependant on the size, number of the brain lesion and proximity to critical structures.

### Regarding patient follow-up

The follow-up included neurologic examinations and magnetic resonance imaging 3 months after start of treatment and in 3 months intervals to evaluate response or failure criteria and to evaluate treatment morbidity.

### Statistical methods

This was an intention to treat analysis.

Comparison between two groups for numerical variables was done using either student t- test or Mann–Whitney –U test (non-parametric t-test) as appropriate. Comparison between more than two groups for numerical variables was done using ANOVA or non parametric Kruskal-Wallis as appropriate. Relation between qualitative data was done using Chi-square test or non- parametric Fisher’s exact test as appropriate. Survival analysis was done using Kaplan-Meier curve with log rank for comparison between different arms.

## Results

The present work included 60 patients with brain metastases divided into 3 arms; 21 patients received *WBRT + SRS*, 21 patients received *WBRT* and 18 patients received *SRS*. The mean follow up duration was 10 months and the median follow up duration was 8.5 months (range 0–34 months).

All the thirty nine patients who received SRS were treated with Brown-Roberts-Wells CT stereotactic (BRW) localization system frame.

The mean prescribed isodose line was 89% (ranged from 85% to 93%). All lesions were treated with prescription isodose volume to target volume ratio (PITV) as classified per our protocol (ranged from 1 to 1.5, mean = 1.3).

Twenty six patients (66.7%) were treated using non coplanar arcs by the cone, with a diameter ranging from 1.25 to 4 cm; the remaining thirteen patients (33.3%) were treated using the mMLC. The number of beams used ranged from 5 to 7 beams with a mean of 6 beams.

### Number of brain lesions

The majority of patients had single metastasis 70% of patients (42/60) with 15 (71.4%), 13 (62%) and 14 (77.8%) in WBRT + SRS, WBRT & SRS arms, respectively, 23.3% of patients had two lesions (14/60) with 5 (23.8%), 5 (23.8%) and 4 (22.2%) in WBRT + SRS, WBRT & SRS arms, respectively while 6.7% of patients (4/60) had three lesions with 1 (4.8%), 3 (14.2%) and 0 (0%) in WBRT + SRS, WBRT & SRS arms, respectively. The total no. of treated brain lesions was 82 with 28 lesions in WBRT + SRS arm, 32 lesions in the WBRT arm and 22 lesions in the SRS arm.

### Largest brain lesion size

Brain lesion(s) with maximum dimensions larger than or equal 3 cm were found in 50% of patients (30/60) with 9 patients (42.8%), 16 patients (76.2%) and 5 patients (28%) in the WBRT + SRS, WBRT & SRS arms, respectively.

### Sites of the brain lesions

Lobar lesions were the most common and were found in 68/82 lesions (82.9%) followed by cerebellar lesions (14/82 lesions i.e., 17.1%).

According to number of brain sites involved; 46 patients (76.7%) had only one site involved (e.g. unilateral parietal lobe, frontal lobe, occipital lobe, temporal lobe, cerebellum), while 14 patients (23.3%) had more than one site involved. Regarding the three treatment arms; 17 patients (80%), 14 patients (67%) and 15 patients (83.3%) had one brain site involved in WBRT + SRS, WBRT & SRS arms, respectively.

#### ***Definition of response***

The bidimensional product for each of the brain metastases identified at baseline was measured and calculated. The bidimensional product is defined as the largest dimension multiplied by the second largest dimension that is perpendicular to it. This value was recorded on the baseline form and every subsequent follow-up form and the response was recorded as whether there is CR or PR or no response or CNS progression. The appearance (yes/no) of any new brain metastases was recorded on all follow-up forms. Local control was defined as unchanged or improved serial post-treatment MRI scans judged as either complete response, partial response, or stable disease.

– **Complete response *(CR****)* - disappearance of all tumors on consecutive CT or MRI scans at least one month apart, off steroids, and neurologically stable or improved.

– **Partial response *(PR)*** - 50% or greater decrease in tumor size on consecutive CT or MRI scans at least one month apart, steroid dose stable or reduced, and neurologically stable or improved.

– **Stable disease** - a 0-50% decrease in size of all lesions with improving or stable neurological examination.

– **CNS progression** - was defined as a defined increase in perpendicular bi-dimensional tumor area for any of the 1–3 tracked brain metastases, or the appearance of any new brain metastasis on a follow-up MRI.

For lesions smaller than 1 cm in maximum diameter, a maximum increase of 50% in perpendicular bi-dimensional treatment area was necessary to score as progression. This caveat was included to account for potential variability in measurement, which was most susceptible to proportionate errors at smaller sizes. For greater than 1 cm lesions, the definition was a 25% rule for change.

#### ***Rates of response***

Review showed non-significant higher response rates at 3 months in the WBRT + SRS group 71.5% of lesions *vs* 34.4% of lesions in the WBRT arm vs 45.4% of lesions in the SRS arm *(P = 0.49).*

#### ***Local control analysis***

**Figure 2:**
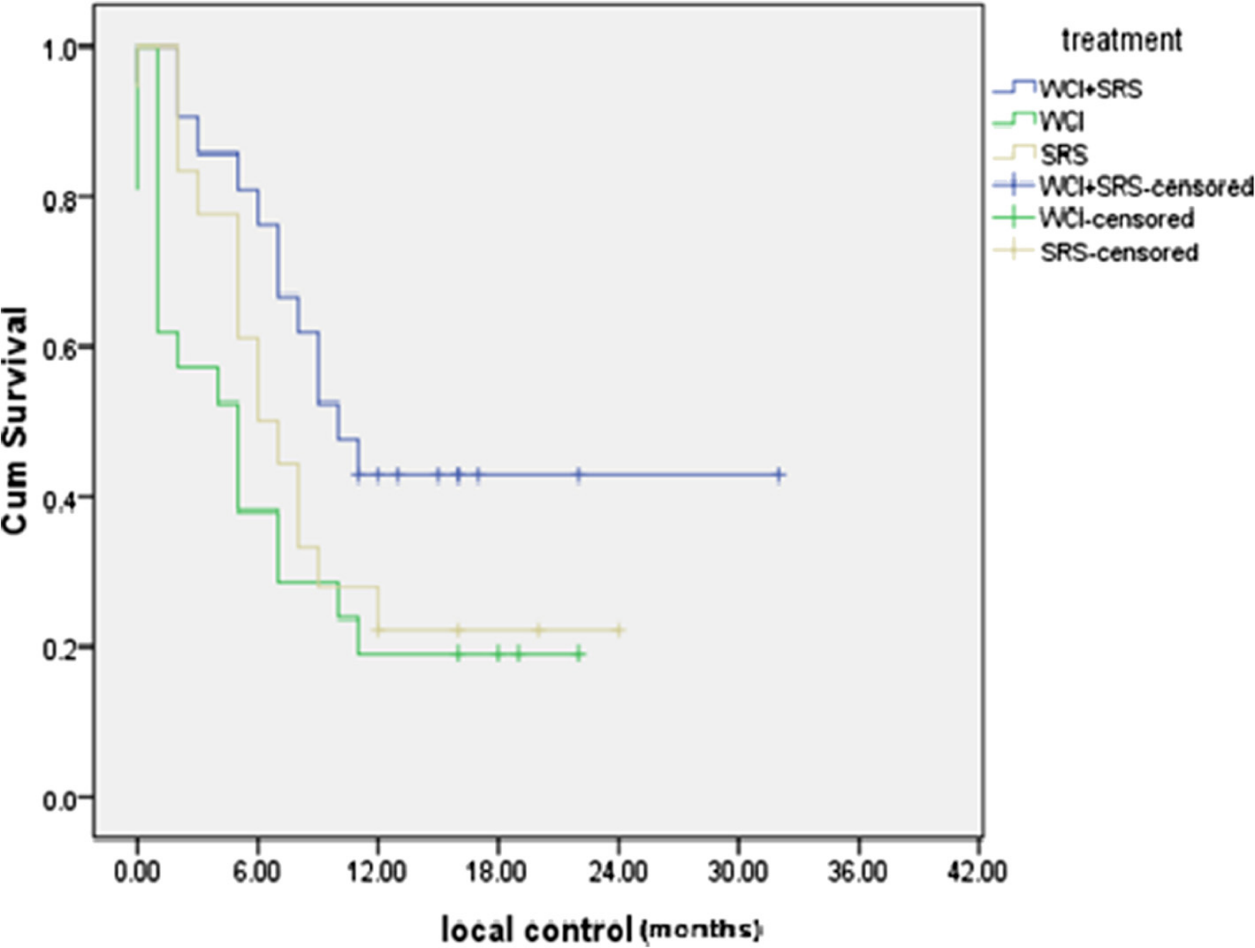
Local control of the 3 treatments groups.

The best local control (LC) of the treated patients at 1 year was reported in the WBRT + SRS group 9/21 (42.9%) *vs* 4/21 (19%) for WBRT group and 4/18 (22.2%) for SRS group with statistically significant *P value = 0.04* as shown in Figure 2*.*

**Figure 3:**
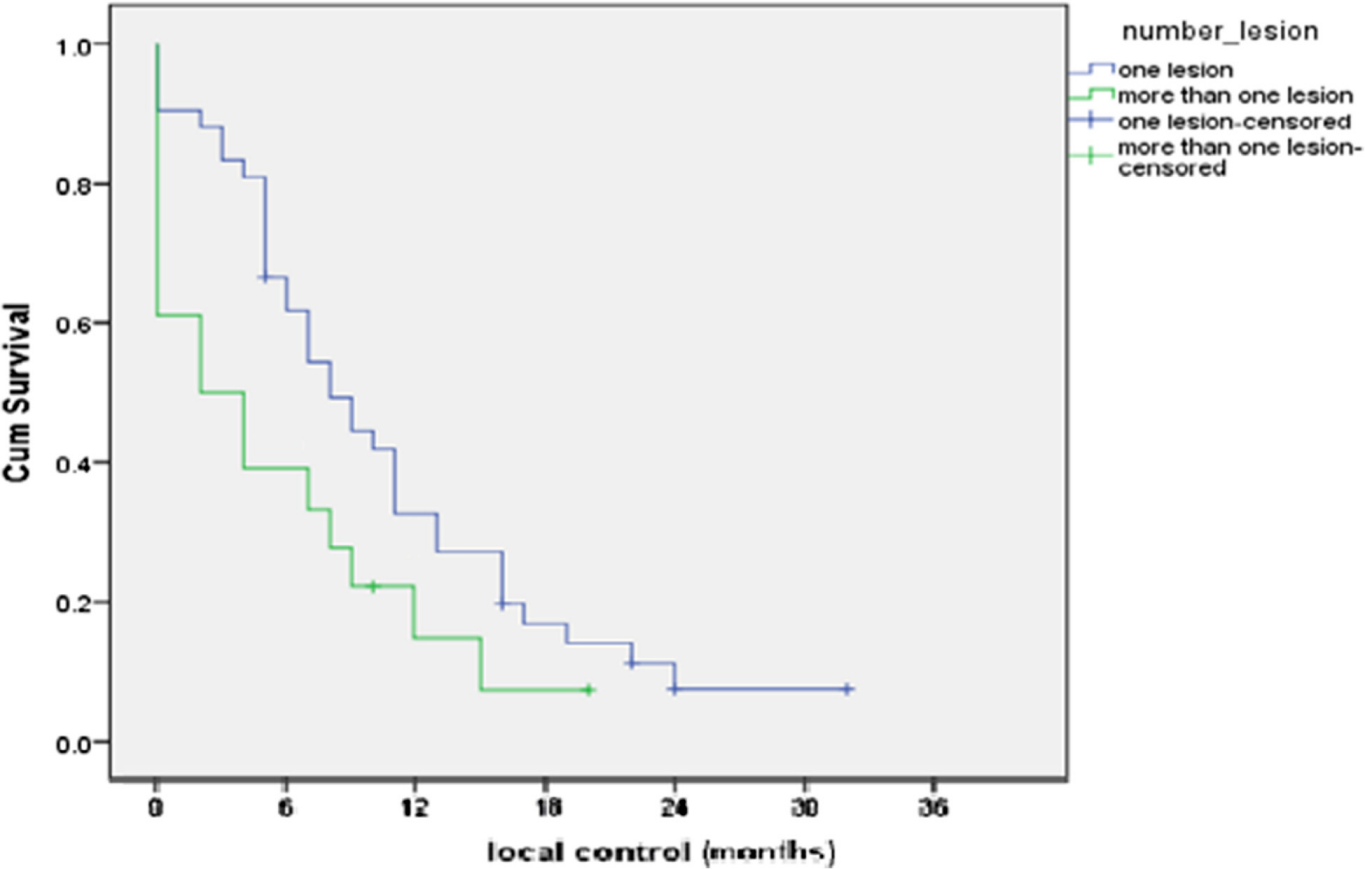
The LC according to number of brain lesions.

As regards number of brain lesions (that is to say the total number of brain lesions regardless their anatomical site) and its effect on LC duration; Figure 3 shows significantly better 6 months LC and 1 year LC duration were reported for patients with single lesion (61.9% and 33.3% respectively) compared to those with multiple lesions (39% and 16.6% respectively) with statistically significant *P value = 0.05.*

**Figure 4:**
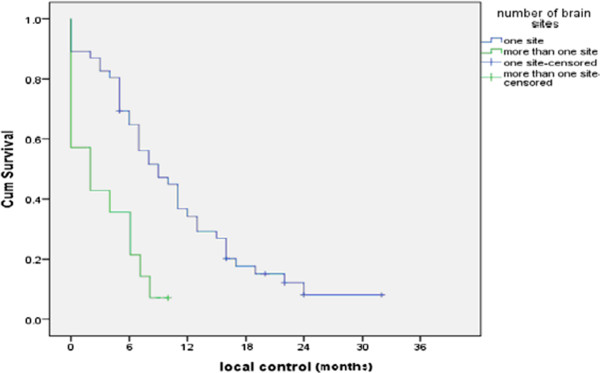
The LC according to number of brain sites involved.

As regard number of sites of brain lesions; (that is to say the anatomical sites of the brain involved by lesions regardless their number) significantly better 6 months LC and 1 year LC duration were reported for patients with lesions in one brain site (65% and 34.7 respectively) compared to those with lesions in multiple brain sites (21.5% and 7.1% respectively) with statistically significant *P value = 0.001* as shown in Figure 4.

Subgroup analysis indicated that WBRT plus SRS provided LC benefit to patients with brain metastasis of less than 3 cm maximum dimension (median LC was 12 vs 3 vs 6 months for WBRT + SRS vs WBRT vs SRS, respectively) with statistically significant *P value = 0.004.*

#### ***Survival analysis***

**Figure 5:**
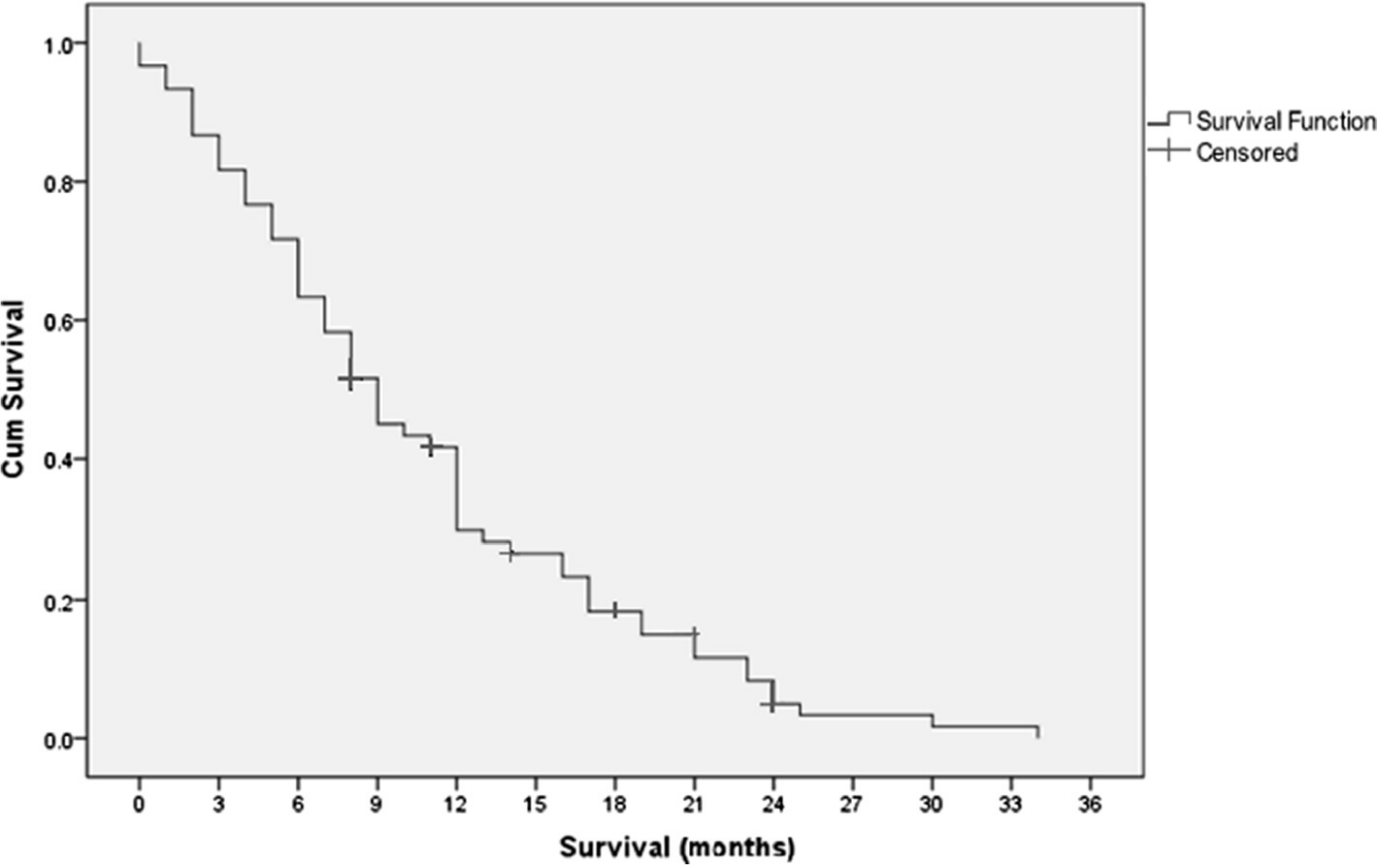
Overall survival for all patients.

**Figure 6:**
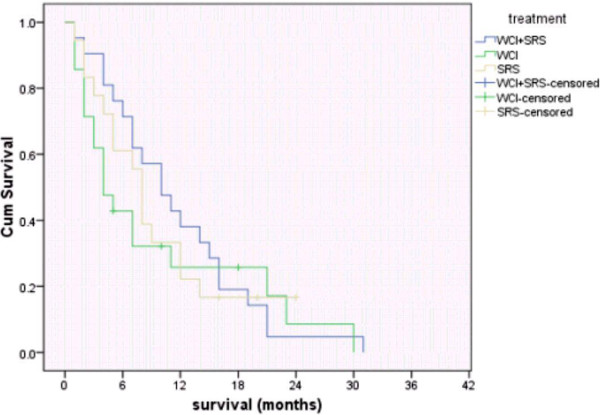
Overall survival for each treatment group.

Figure 5 shows the median survival time for all patients was 8.5 month. Overall survival at 6 month was 61.6% and at 12 month was 30%. While Figure 6 shows overall survival according to the treatment groups.

**Figure 7:**
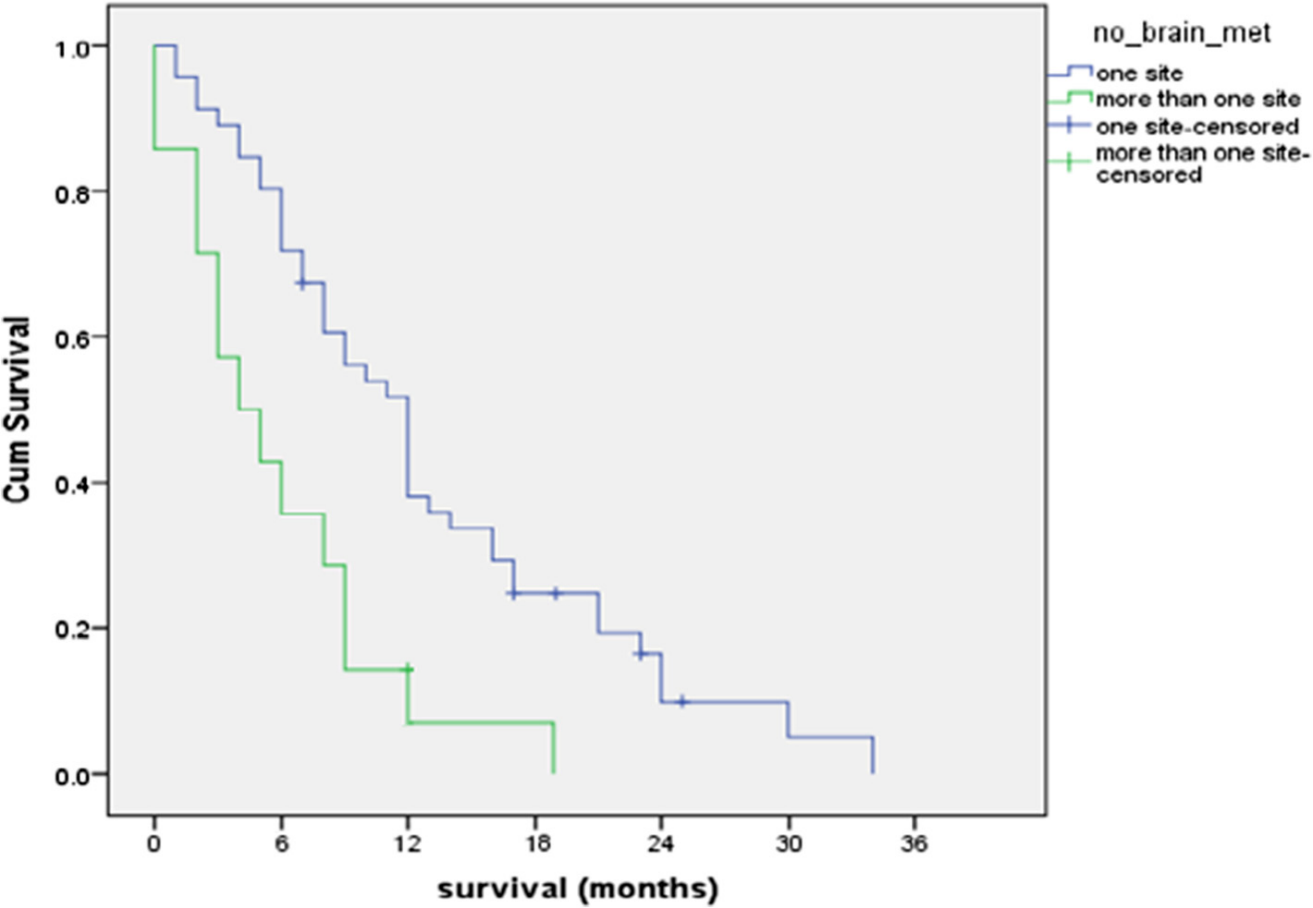
Overall survival according to number of brain sites involved.

As regard number of sites of brain lesions; significantly better 6 months, 1 year were reported for patients with lesions in one brain site (69.5%, 37% respectively) compared to those with lesions in multiple brain sites (35.7% and 7.1% respectively) *(P = 0.003) *as shown in Figure 7*.*

Subgroup analysis indicated that WBRT plus SRS provided survival benefit to patients whose largest brain metastasis was 3 cm in diameter (median survival was 15 months vs 8 months vs 5 months for WBRT + SRS vs SRS vs WBRT, respectively with statistically significant *P value = 0.002*), also subgroup analysis showed that patients with controlled primary who recieved WBRT plus SRS had survival benefit compared to SRS vs WBRT (median survival was 12 months vs 8 months vs 5.5 months for WBRT + SRS vs SRS vs WBRT, respectively with statistically significant *P value = 0.027*).

Overall survival was compared with different prognostic factors such as treatment modality recieved, number of lesions , if any previous chemotherapy was given and the RPA but all these correlations were statistically insignificant.

#### ***Treatment related toxicity***

Acute toxicities were identified as events that arose within 90 days from start of radiotherapy and late toxicities as events that occurred 3 months after start of radiotherapy and thereafter. Acute and late toxicities did not differ greatly between treatment groups [Tables 1 and 2].

**Table 1:** Number of patients suffering from treatment-related acute toxicity

	**WBRT + SRS**	**WBRT**	**SRS**
> Grade 2 headache	2	2	2
> Grade 2 vomiting	0	0	1
> Grade 2 radiation dermatitis	0	0	0
Neurologic worsening (without CNS progression)	2	1	0
Seizures	0	0	1 (grade 2 seizures)
Total	3 (14%)	3 (14%)	2 (11%)

**Table 2:** Number of patients suffering from treatment-related late toxicity

	**WBRT + SRS**	**WBRT**	**SRS**
Radionecrosis	1	0	1
Brain oedema	1	1	1
Neurologic worsening (without CNS progression)	2	1	2
Total	3 (14%)	2 (9%)	2 (11%)

The total number of patients was less than the calculated total as some patients had multiple complications. Grading is according to the National Cancer Institute’s Common Toxicity Criteria version 2.0 (Trotti et al., 2000).

The total number of patients was less than the calculated total as some patients had multiple complications. Neurologic worsening is classified according to the RTOG/EORTC late radiation morbidity scoring schema (RTOG, 1999).

## Discussion

### Regarding local control

The current study reported improvement in local control in patients treated with WBRT + SRS as compared to WBRT alone and to SRS alone, where the median local control was 10 months for WBRT + SRS compared to 6 months and 5 months for SRS alone and WBRT alone, respectively *(P = 0.048).*

These results are similar to Kondziolka et al., Chougule et al. and Andrews et al., these three randomized trials that detected an improvement in local brain control in patients treated with WBRT + SRS as compared with WBRT alone [9-11]. Also these results are similar to the results of Aoyama et al., Chougule et al., Chang et al. and Kocher et al. [12]. These four randomized trials that detected an improvement in local brain control in patients treated with WBRT + SRS as compared with SRS alone [5,10,13,14].

As regard response rate; MRI assessment showed higher response rates of brain lesions at 3 months follow up for WBRT + SRS (71.5%) compared to 45.4% and 34.4% for SRS alone and WBRT alone , respectively *(P = 0.49).* This result is similar to that of the RTOG-9508 trial which revealed higher response rate at 3 months follow-up for WBRT + SRS compared to WBRT alone after assessing 153 MRI sets out of 270 MRI sets for 270 surviving patients *(P = 0.043)*[11]*.*

### Regarding overall survival

The current study revealed no significant difference in 6-month, 12-month and median survival between the three treatment arms.

These results are similar to Kondziolktout et al., Chougule et al. and Andrews et al., were these three randomized trials reported non statistically significant improvement in overall survival with the use of WBRT and SRS boost as compared with WBRT alone [9-11].

In the current study analysis of the different prognostic factors that might affect the overall survival in current study were done; the current study reported a highly significant better 6 months, 1 year survival & median survival duration for patients with lesions in one brain site compared to those with lesions in multiple brain sites *(P = 0.003).*

Subgroup survival analyses of the RTOG-9508 trial revealed a survival advantage in the WBRT + SRS group for patients with a single brain metastasis *(p = 0.039),* also there were trends towards better survival with SRS boost in patients with largest tumor > 2 cm, favourable histology, KPS ≥90 & RPA class 1 patients [11]*.*

### Regarding SRS doses

The prescribed doses in the WBRT + SRS arm in our study were less than those prescribed in the RTOG-9508 trial which treated metastases ≤ 2 cm in maximum diameter with a dose of 24 Gy; metastases > 2 cm to 3 cm with 18 Gy; and metastases > 3 cm to 4 cm with 15 Gy [11], this was due to several reasons:

(a) The lack of sufficient of experience regarding SRS in our department being a new technique for us with fear of increased complications with higher doses.

(b) Our doses were in line with those used in the JROSG 99–1 trial which treated metastases with a maximum diameter of ≤ 2 cm with 22 to 25 Gy and those with 2–3 cm with 18 to 20 Gy in the SRS alone arm. These doses were reduced by 30% when the treatment was combined with WBRT. The JROSG 99–1 trial reported that these choices of doses were due to “our preexisting experience of SRS with a 30% reduced SRS dose combined with WBRT indicated that there is not a significant difference in local tumor control (data not shown) compared with SRS with the dose suggested in the RTOG protocol” [5].

(c) The RTOG-9508 trial reported “no survival advantage was noted between groups when assessing dose delivered” and also “higher isodose prescriptions did not affect local control rates in the radiosurgery boost arm” [11]*.*

## Conclusions

In conclusion, our data suggest that WBRT + SRS is an effective, safe tool in treatment of patients with 1 to 3 brain metastases improving the local control compared to WBRT alone and SRS alone especially for patients with brain metastasis of less than 3 cm maximum dimension and with acceptable tolerance especially regarding neurological toxicity. Also WBRT + SRS provide better survival in patients whose largest brain metastasis was less 3 cm in diameter and those with controlled primary. Regardless of the treatment group, our data suggest that both survival and local control are better for single brain site involvement compared to multiple brain sites involvement. Further studies with larger number of patients is needed to be conducted for more reliable statistical results.

## Competing interests

The authors declare that they have no competing interests.

## Authors’ contributions

All authors contributed in all steps of the study they were working together and completing each other and all authors read and approved the final manuscript.
